# Use of real time continuous glucose monitoring and intravenous insulin in type 1 diabetic mothers to prevent respiratory distress and hypoglycaemia in infants

**DOI:** 10.1186/1471-2393-8-23

**Published:** 2008-07-01

**Authors:** Dario Iafusco, Fabrizio Stoppoloni, Gennaro Salvia, Gilberto Vernetti, Patrizia Passaro, Goran Petrovski, Francesco Prisco

**Affiliations:** 1 Department of Paediatrics, Second University of Naples, Italy; 2 Maternal-Fetal Medicine Unit, Buon Consiglio Fatebenefratelli Hospital, Naples, Italy; 3 Neonatology and N.I.C.U. , Buon Consiglio Fatebenefratelli Hospital, Naples, Italy; 4 Clinic of Endocrinology and Diabetes, Skopje, Former Yugoslav Republic of Macedonia

## Abstract

**Background:**

Pregnancy in Type 1 diabetic patients is a precarious condition, both for mother and fetus with increased the risk of prematurity and, immediately after delivery with risk of respiratory distress syndrome and hypoglycaemia in newborns. A strict control and monitoring of diabetes throughout pregnancy is important in reducing the impact of the disease on the fetus and newborn. In recent years many new technologies have been introduced to ameliorate diabetes monitoring, where the last is the Real-time Continuous Glucose Monitoring System (RT-CGMS).

**Methods:**

In the last three years, 72 h continuous glucose monitoring system (RT-CGMS) (Medtronic, CA) was performed in 18 pregnant women with Type 1 diabetes in two moments of pregnancy: during treatment with betamethasone to prevent respiratory distress and during delivery. In both cases insulin was administered intravenous and the dose was changed on the basis of glycaemia.

**Results:**

The results present the use of this new technique during two topics moments of pregnancy of type 1 diabetes patients when is very important intensively to monitor diabetes and to obtain the well being of the fetus. No infant experimented hypoglycaemia or respiratory distress syndrome at the moment and in the first hours after the birth.

**Conclusion:**

We wish to stress the importance reducing glycaemia during administration of betamethasone and during labor. It is conceivable that the scarce attention paid to monitoring glucose levels in diabetic mothers during labor in gynaecological world may be due to the difficulty in glucose monitoring with the devices until now available. Hopefully, our anecdotal account may prompt improvements with RT-CGMS, and may lead to a better approach to the problem, thereby changing the prognosis of infants born to diabetic mothers.

## Background

Infants born to type 1 diabetic mothers are at significantly greater risk for perinatal morbidity. Two conditions are very frequent at the birth: respiratory distress syndrome (RDS) and hypoglycaemia. Strict maternal glycemic control during pregnancy complicated by diabetes mellitus reduces neonatal morbidity and mortality.

### Respiratory Distress Syndrome

Infants of type 1 diabetic mothers are more likely to have respiratory symptoms in the newborn period from either RDS (surfactant deficiency) or retained fetal lung fluid (transient tachypnea of the newborn) after operative delivery.[1]. RDS occurs more frequently in IDMs (Infants of Diabetic Mother) because of later onset of maturity of the type II alveolar cells[2] and is secondary to pulmonary surfactant deficiency. Fetal hyperinsulinism is a key factor in the pathogenesis of RDS because insulin is believed to antagonize the physiological maturing effect of cortisol. [3]. Hyperinsulinism is also responsible for polycythemia, a condition inducing persistent pulmonary hypertension which complicates the course of RDS.

Ideally, RDS is prevented by excellent maternal glycemic control during pregnancy [2]. Corticosteroids are strongly recommended to prevent prematurity complications in newborns of non-diabetic mothers in whom a rise in blood glucose levels in the two days following administration of betamethasone has been reported [4]. Due to the higher risk of RDS in infants from diabetic mothers administration of betamethasone to the mother is even more advisable then in non-diabetic mothers. On the other side the consequent rise in blood glucose levels [4] is expected to be more marked in diabetic than in non-diabetic mothers thus requiring a strict adjustment of the insulin therapy. This because the fetal hyperinsulinism consequent to the maternal hyperglycemic peak could block the beneficial effect of the administered betamethasone on fetal pulmonary maturation.

### Hypoglycaemia

In the diabetic pregnant the stress during the labour usually induces a further increase of blood glucose levels with a consequent rise of the fetal production of insulin and increased risk of hypoglycemia. Therefore, a close monitoring of blood glucose in the mother together with an appropriate insulin treatment during the last hours before delivery are needed.

Over the past years practitioners have sought to improve the outcome of diabetic pregnancies. In pregnancies complicated by type 1 diabetes, where excellent glucose control is desired to improve maternal and fetal outcomes, RT-CGMS, a novel well tolerated tool to assess 24-h glucose fluctuations, may have a role in fine-tuning management [5]. Its role may be crucial in two topic moments of pregnancy such as betamethasone therapy and labor.

## Methods

Eighteen pregnant women (mean age 23.4 ± 2.5 yrs; range 18–28 yrs) with type 1 diabetes (mean age at the diagnosis 8.5 ± 3.3 yrs; mean duration of the disease 14.8 ± 2.9 yrs) were consecutively enrolled in the study in the last 3 years (2004–2007).

The research plan has been approved by the Ethics Committee of Department of Pediatrics of the Second University of Naples. An informed consent was signed by all mothers taking part to the study.

All patients wore Real Time Continuous Glucose Monitoring System sensors (RT-CGMS), in two moments: during treatment with betamethasone and in the perinatal period and during labor. The glycemic profile was obtained with a continuous glucose monitoring system, not equipped for real-time visualization of the results (CGMS Medtronic), in the first 4 patients at the beginning of the study and with a Guardian^® ^Real Time CGMS (Medtronic) equipped for real-time visualization of the results in the others.

The CGMS unit consists of a glucose sensor, which is inserted into the subcutaneous tissue of the body and left in place for up to 72 hours. This sensor senses the interstitial fluid glucose levels electrochemically every 10 seconds, records an average value every 5 minutes and gives 288 values per day. The sensor of CGMS after 72 hours is removed and the data from the monitor are downloaded into a PC, which gives a continuous graph of the glucose values of the previous 3 days; Guardian^® ^Real Time CGMS (Medtronic) uses a continuous telemetry display of real-time glucose values.

The RT-CGMS is an accurate tool for additional glucose monitoring in pregnant women with type 1 diabetes mellitus as previously demonstrated [6].

Betamethasone was administered intramuscularly (12 mg/24 h for two days) between the 30^th ^and 32^nd ^week of gestation.

Continuous intravenous infusion of insulin (between 0.02 U/kg/h and 0.06 U/kg/h), guided by glucose levels, enabled us to reach and maintain glucose levels constant between 100 and 150 mg/dl during treatment with betamethasone and during labor up to delivery in the vaginal delivery and between 80 and 100 mg/dl during the caesarean section birth.

## Results and Discussion

At the beginning of the study after betamethasone administration we tried to maintain the multi-injection insulin therapy but it did not prevent an increase of glycaemia despite of the increasing insulin dose.

**Figure 1:**
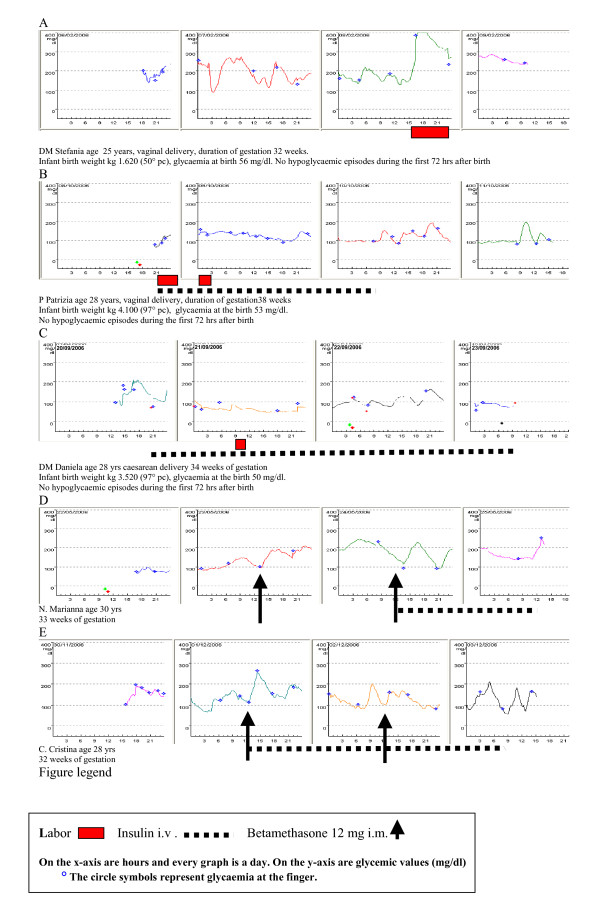
Continuous Graph of the glucose values of 3 days.

Fig 1D shows the continuous graph of the glucose values of a patient who received, the day after betamethasone, multi-injection insulin therapy at a dose of 0,9 Units/Kg/day in 4 administrations/day, which did not prevent an increase of glycaemia monitored for about 24 h (around 200 mg/dl with a peak of 250 mg/dl after 15 h). After the second betamethasone injection, intravenous insulin was introduced reducing the degree and duration of hyperglycaemic peaks (<200 mg/dl).

Fig. 1E shows the graph of the first patient in which intravenous insulin was introduced after the first betamethasone dose resulting in an acceptable level of glycaemia from the beginning and for all two days of treatment.

We wish to stress the importance of reducing glycaemia and keeping blood glucose under tight control during administration of betamethasone not only for the metabolic status of the mother but to avoid hyperinsulinism of fetus that block the beneficial effect of betamethasone on pulmonary maturation.

No newborn from mothers of our study developed Respiratory Distress Syndrome after the birth.

Another period of high risk for babies from type 1 diabetic mothers is during delivery, when hyperglycaemia could induce the newborns to produce high amounts of insulin with the consequence of hypoglycaemic status after the cut of the blood cord.

The profile in Figure 1A, obtained with a continuous glucose monitoring system (CGMS^®^; Medtronic USA) not equipped for real-time visualization of the results, shows that rapid, prolonged hyperglycaemia can occur during labor and has provided opportunities to examine limitations of conventional monitoring of glycaemia by intermittent finger-stick testing.

Figure 1B and 1C (vaginal delivery and caesarean section) obtained with a Guardian^® ^Real Time CGMS (Medtronic) equipped for real-time visualization of the results shows that the continuous infusion of insulin (between 0.02 U/kg/h and 0.06 U/kg/h), guided by glucose levels, enabled us to reach and maintain glucose levels constant between 100 and 150 mg/dl during labor up to the vaginal delivery and between 80 and 100 mg/dl during the caesarean section birth.

The mean glycemic values of infants immediately after birth was 84 ± 16 mg/dl and no hypoglycaemic episode was recorded during the first 72 hrs after birth.

## Conclusion

Real-time CGMS is a very useful tool for obstetrics and diabetologists during the follow up of the pregnant type 1 diabetic patients in particular when the objective of the therapy is euglycaemia. The sensor is useful because it permits a closer observation of the fluctuation of blood glucose levels. It would be impossible to measure blood glucose on capillary blood at the same frequency intervals mainly for the discomfort of the repeated punctures.

Therefore, we wish to stress the importance of reducing glycaemia during administration of betamethasone and during the labor. It is conceivable that the scarce attention paid to monitoring glucose levels in diabetic mothers during labour in gynaecological worlds may be due to the difficulty in glucose monitoring with the traditional devices until now available. Hopefully, our anecdotal account may prompt improvements in CGMS, and may lead to a better approach to the problem, thereby changing the prognosis of infants born to diabetic mothers.

## Competing interests

The authors declare that they have no competing interests.

## Authors' contributions

DI conceived the study, and participated in its design and coordination. FS participated in the study design and coordination. GS participated in the design of the study and performed the analysis of the results. GV participated in the design of the study. PP have made substantial contributions to acquisition of data. GP have been involved in drafting the manuscript and revising it critically for important intellectual content. FP conceived the study, and participated in its design and interpretation of data. All Authors read and approved the final manuscript.

## Pre-publication history

The pre-publication history for this paper can be accessed here:


